# Surface code for low-density qubit array

**DOI:** 10.1038/s41598-022-17090-6

**Published:** 2022-07-28

**Authors:** Tatsuya Tomaru, Chihiro Yoshimura, Hiroyuki Mizuno

**Affiliations:** Center for Exploratory Research, Research and Development Group, Hitachi, Ltd., Kokubunji, Tokyo 185-8601 Japan

**Keywords:** Quantum information, Qubits

## Abstract

Surface code is a promising candidate for the quantum error corrections needed for fault-tolerant quantum computations because it can operate on a two-dimensional grid of qubits. However, the gates and control lines become dense as more and more qubits are integrated, making their design and control difficult. This problem can be alleviated if the surface code can operate on sparse qubit arrays. Here, we give an solution for an array in which qubits are placed on edges as well as on nodes of a two-dimensional grid. The qubits on the edges are divided into two groups: those in one group act as the deputies of data qubits; the others act as deputies of the syndrome qubits. Syndrome outputs are obtained by multiplying the measured values of the syndrome and edge qubits. The procedure for the quantum part is the same as that of the ordinary surface code, making the surface code applicable to sparse qubit arrays.

## Introduction

Quantum error correction is essential to make fault-tolerant quantum computation a reality. Surface code, which originated from toric code^1,2^, is a promising candidate because the threshold error rate is relatively high, ~ 1%^3–7^, and because it can operate on a two-dimensional grid of qubits^8^, which seems to fit practical device architectures. However, the implementation is not so simple. Each qubit must be individually controlled; therefore, how to implement the gates and control lines becomes an issue because there is little space in the highly integrated device. In addition, it is preferable for all qubits to be measurable, if possible. Moreover, two-qubit interactions need to be switched on only during the two-qubit gate; at other times, the interactions must be off. Parallel gate operation is also needed, and cross-talk should be suppressed. It is not easy to satisfy all these requirements. If qubits are loosely laid out with a large inter-qubit distance, the problem might be resolved. However, the large distance weakens a direct inter-qubit interaction in qubit systems such as semiconductor ones. In addition, the loose layout goes against the target that densely integrating qubits is one of the milestones for making large-scale fault-tolerant quantum computing a reality.

As for integrating qubits, various architectures have been proposed for semiconductor qubit arrays that have high potential for integration. One is a three-dimensional architecture^9^. Another is that two quantum dots are used as the position of one spin qubit^10^. These proposals do not seem to be easy to implement. One reason for the difficulty comes from the geometrical constraint regarding the surface code; the constraint makes the architecture in the proposals rather complicated as a consequence. Here, if the surface code can operate on a sparser qubit array, the difficulty will be reduced because unused space can be allocated to control regions; thereby, freedom in design is increased; the gates and control lines can be more flexibly laid out; the strength of the inter-qubit interaction is maintained in this case; and crosstalk is reduced because the number of nearest-neighbor qubits is decreased.

As a solution to utilize the merits, Buonacorsi et al. proposed an architecture of shuttling qubits, in which units with four qubits are sparsely arranged and the units are connected with shuttle lines^11^. Boter et al. proposed a spider-web array where two-qubit interaction parts are sparsely arranged and qubits are shuttled between the interaction parts^12^.

This report gives another solution. We propose a low-density qubit array and a surface-code operation on it. The array consists of qubits placed on edges as well as on nodes of the two-dimensional grid. One group of edge qubits works as deputies of the data qubits; the other group works as deputies of the syndrome qubits. The stabilizer measurements are performed using CNOT operations between the data qubits and deputy syndrome qubits and between the deputy data qubits and syndrome qubits, instead of between the data and syndrome qubits in ordinary surface code. Therefore, the procedure for the quantum part of our stabilizer measurements is the same as that of ordinary surface code. The difference is that syndrome outputs are obtained by multiplying the measured values of the syndrome qubits and the edge qubits. Multiplying the measured values corresponds to feedforward^13,14^; it is not based on subsystem codes such as Bacon–Shor code^15,16^.

Despite having a low-density qubit array, the procedure for the quantum part in our method is the same as that of ordinary surface code; therefore, the cycle of the stabilizer measurements is held to a minimum, although the procedure for the classical part increases. Thus, the surface code can operate on a low-density qubit array, reducing the difficulty in designing and controlling the array.

## Results

### Layout and stabilizers

The surface code for a low-density qubit array can be understood smoothly by referring to the ordinary surface code. Therefore, we first mention the ordinary surface code briefly.

**Figure 1:**
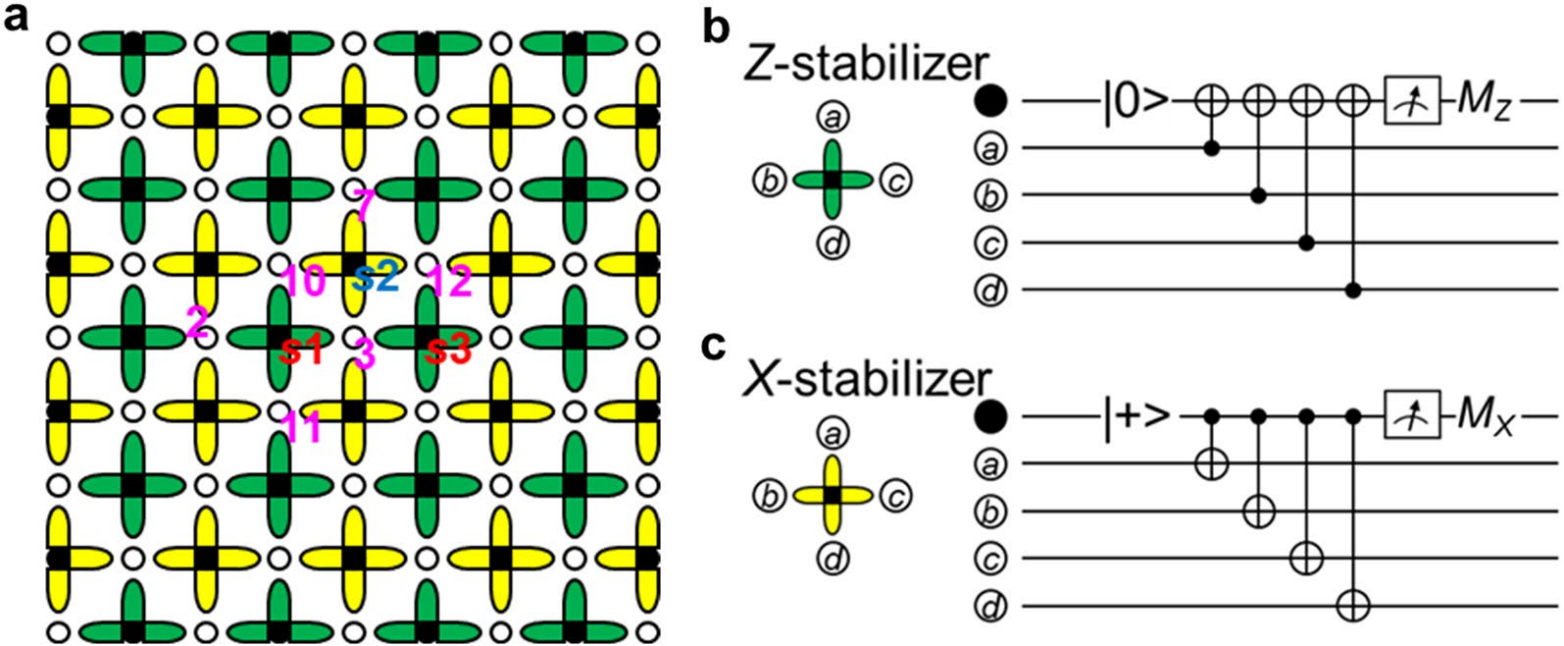
Schematic surface code. (**a**) Open circles show data qubits with information; solid circles show syndrome qubits that are ancillas. Data qubits interact with syndrome qubits through green and yellow edges. The syndrome qubits surrounded by green edges are assigned to detecting a bit-flip error; those surrounded by yellow edges are assigned to detecting a phase-flip error. Pink numerals indicate the positions of data qubits. Red and blue symbols starting “*s*” indicate the positions of syndrome qubits. (**b**) Circuit for detecting a bit-flip error. (**c**) Circuit for detecting a phase-flip error.

Figure 1a shows a schematic view of the surface code, which is a stabilizer code^17,18^. Physical qubits are grouped into data qubits, in which quantum information is stored, drawn with open circles, and syndrome qubits, which are used to detect and stabilize data qubits, drawn with solid circles. As can be seen, the data and syndrome qubits are alternately arranged on a two-dimensional grid. Each syndrome qubit is surrounded by four data qubits that compose a stabilizer, except for boundaries. Let $\hat{X}_{k}$, $\hat{Y}_{k}$, and $\hat{Z}_{k}$ be the Pauli operators for site *k*. The stabilizers are $\prod\nolimits_{i} {\hat{Z}_{i} }$ and $\prod\nolimits_{j} {\hat{X}_{j} }$, where *i* denotes the data qubits connected to one of the syndrome qubits surrounded by the green edges in Fig. 1a, and *j* denotes the data qubits connected to one of the syndrome qubits surrounded by the yellow edges. The green and yellow edges are alternately aligned in rows and columns.

Figure 1b shows the circuit for the *Z*-stabilizer measurements. The syndrome qubit is initialized to |0>; the information of the data qubits is transferred to the syndrome qubit through CNOTs; and the syndrome qubit is measured in the *Z*-basis. This cycle is repeated. When a bit-flip error does not occur at any qubits of *a*, *b*, *c*, and *d*, the syndrome output does not change between consecutive measurements; i.e., the *Z*-stabilizer measurements can detect a bit-flip error. Figure 1c shows the circuit for the *X*-stabilizer measurements. The initialization to |0> in Fig. 1b is changed to |+> = (|0> +|1>), where a normalization factor is omitted and will be similarly omitted hereafter; control and target qubits are interchanged; and the measurement is changed from the *Z*-basis to the *X*-basis. The *X*-stabilizer measurements can detect a phase-flip error. The stabilizer measurements shown in Fig. 1b and c are performed for all stabilizers simultaneously and repeatedly.

All stabilizers commute. This can be checked by considering adjacent *Z*- and *X*-stabilizers; the commutation of the other pairs is trivial. Let $\hat{Z}_{s1} \equiv \hat{Z}_{2} \hat{Z}_{3} \hat{Z}_{10} \hat{Z}_{11}$ and $\hat{X}_{s2}$≡ $\hat{X}_{3} \hat{X}_{7} \hat{X}_{10} \hat{X}_{12}$ by referring to Fig. 1a. The common sites between $\hat{Z}_{s1}$ and $\hat{X}_{s2}$ are 3 and 10; $\hat{Z}_{s1}$ and $\hat{X}_{s2}$ commute because $\left[ {\hat{Z}_{i} \hat{Z}_{j} ,\hat{X}_{j} \hat{X}_{i} } \right]$ = 0.

**Figure 2:**
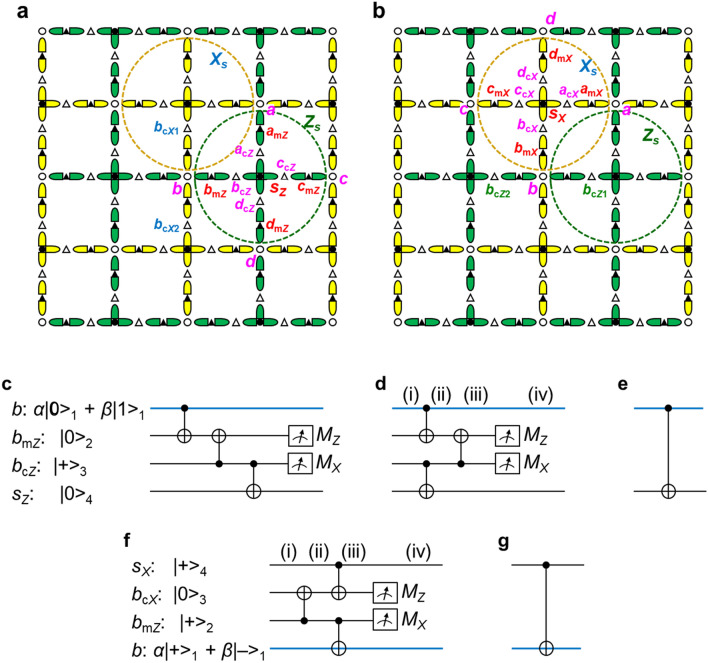
Qubit array where qubits are located on each edge as well as on nodes of the grid. (**a**,**b**) Open and black circles respectively are data and syndrome qubits, which are configured on the nodes, similar to the ordinary surface code. Qubits indicated by black triangles work as a mediator that transfers the information on a data qubit to a copy qubit indicated with an open triangle. Dashed circles are a guide to visualize data qubits belonging to a stabilizer; green and yellow dashed circles show *Z*- and *X*-stabilizers, respectively. Symbols are mainly allocated for a *Z*-stabilizer in (**a**) and for an *X*-stabilizer in (**b**). (**c**) Circuit for an operation equivalent to CNOT with qubits *b* and *s*_*Z*_ as a control and a target, respectively; the information on *b* is transferred to a copy qubit *b*_c*Z*_ with the help of a mediator qubit *b*_m*Z*_; in place of *b*, *b*_c*Z*_ performs a CNOT operation with *s*_*Z*_. This series of operations is completed with the measurements of *b*_m*Z*_ and *b*_c*Z*_. (**d**) Circuit equivalent to (**c**). (**e**) Circuit equivalent to (**c**) and (**d**) under the feedforward being done in (**c**) and (**d**). (**f**,**g**) Similar to (**d**) and (**e**), but the bases for the input state are replaced with |+> and |−> .

The motivation of this report is to reduce the density of qubits. To achieve it, let us set qubits on edges in addition to on nodes of the two-dimensional grid as shown in Fig. 2a. The qubits on the nodes are the data and syndrome qubits (open and solid circles), similarly to Fig. 1a. The definitions of *X*- and *Z*-stabilizers are similar; each syndrome qubit treats the four nearest data qubits. For example, the yellow and green dashed lines in Fig. 2a outline *X*- and *Z*-stabilizers, where data qubits *a* and *b* are common. Let us call the qubits on each edge the copy qubit (open triangle) and mediator qubit (solid triangle). The naming comes from that the information on a copy qubit is transferred from a data qubit with mediation by a mediator qubit.

The data and syndrome qubits in Fig. 2a are not located at a nearest-neighbor site of the other, different from Fig. 1a; the information on the data qubits cannot be directly transferred to the syndrome qubits. Instead, the information is transferred in the order of, e.g., *b* → *b*_m*Z*_ → *b*_c*Z*_ → *s*_*Z*_ by using the qubits on the edge. The circuit for the transfer is shown in Fig. 2c, and it is redrawn in Fig. 2d because the second and third CNOTs in Fig. 2c are commutable. Qubits *b*_m*Z*_ and *b*_c*Z*_ are initialized to |0> and |+>, respectively; they are measured after the three CNOT operations; the transfer process from *b* to *s*_*Z*_ is completed at the measurements and feedforward. The details of this transfer process are described in the following.

### Principle of information transfer

In ordinary surface code, data and syndrome qubits (open and solid circles, respectively) are alternately arrayed as depicted in Fig. 1a. In this sense, the copy qubits (open triangles) in Fig. 2a take a seat of a data qubit (open circle) and the mediator qubits (solid triangles) take a seat of a syndrome qubit (solid circle). This correspondence also holds true for stabilizer measurements, where copy qubits play the role of data qubits and mediator qubits play the role of syndrome qubits. The order of CNOTs in stabilizer measurements is in accordance with those in Fig. 1b and c, and all stabilizer measurements are synchronized. Accordingly, the timing of CNOT between the *b* − *b*_m*Z*_ sites is the same as that between the *b*_c*Z*_ − *s*_*Z*_ sites. The transform from Fig. 2c to Fig. 2d is for fitting the timing of CNOTs into the order rule. When qubits are set on edges, the number of steps in transferring information from data qubits to syndrome qubits increases. However, thanks to this transformation, CNOTs are executed in parallel; thereby, the circuit depth in the stabilizer measurements is the same as those in Fig. 1b and c.

Let us clarify that the circuit of Fig. 2d transfers the quantum state of *b* to *s*_*Z*_. Let the input state of *b* be *α*|0> _1_ + *β*|1> _1_. Let *b*_m*Z*_, *b*_c*Z*_, and *s*_*Z*_ be initialized to |0> _2_, |+> _3_, and |0> _4_, respectively. The state at position (i) in Fig. 2d is

1 $$\left( {\text{i}} \right){:}\quad (\alpha{|}0 {>}_{1} + \beta\left| {1 {>}_{1} )} \right|0 {>}_{2} \left( {\left| {0 {>}_{3} + } \right|1 {>}_{3} } \right){|}0 {>}_{4} .$$

By using the CNOTs in the first stage, the state is

2 $$\left( { {\text{ii}}} \right){:}\quad (\alpha{|}00 {>}_{12} + \beta\left| {11 {>}_{12} )\;(} \right|00 {>}_{34} + {|}11 {>}_{34} ).$$

By using the CNOT in the second stage, the state is

3 $$\begin{aligned} \left( {{\text{iii}}} \right){:}\quad & (\alpha{|}00 {>}_{12} + \beta\left| {11 {>}_{12} )} \right|00 {>}_{34} + \, (\alpha{|}01 {>}_{12} + \beta\left| {10 {>}_{12} )} \right|11 {>}_{34} \\ & \quad = (\alpha{|}00 {>}_{12} + \beta\left| {11 {>}_{12} )\;(} \right| + {>}_{3} + \, \left| {{-} {>}_{3} )} \right|0 {>}_{4} + \, (\alpha{|}01 {>}_{12} + \beta\left| {10 {>}_{12} )\;(} \right| + {>}_{3} {-} \, \left| {{-} {>}_{3} )} \right|1 {>}_{4} . \\ \end{aligned}$$

*b*_m*Z*_ is measured in the *Z*-basis, and *b*_c*Z*_ is measured in the *X*-basis. The state at position (iv) depends on the measured values (*M*_*Z*_, *M*_*X*_):

4 $$\begin{aligned} \left( {{\text{iv}}} \right){:}\quad & \alpha{|}00 {>}_{{{14}}} + \beta{|11} {>}_{{{14}}} \quad\;\; {\text{for}}\;\left( { + {1}, \, + {1}} \right) \\ & \alpha{|}00 {>}_{{{14}}} {-}\beta{|11} {>}_{{{14}}} \quad\;\; {\text{for}}\;\left( { + {1}, \, {-}{1}} \right) \\ & \alpha{|}0{1} {>}_{{{14}}} + \beta{|1}0 {>}_{{{14}}} \quad\;\; {\text{for}}\;\left( {{-}{1}, \, + {1}} \right) \\ & {-}\alpha{|}0{1} {>}_{{{14}}} + \beta{|1}0 {>}_{{{14}}} \quad {\text{for}}\;\left( {{-}{1}, \, {-}{1}} \right). \\ \end{aligned}$$

When *M*_*Z*_ = + 1 and *M*_*X*_ = + 1, the result is equal to that for the circuit in Fig. 2e. When *M*_*X*_ = − 1, phase-flip *s*_*Z*_ as a feedforward, and when *M*_*Z*_ = − 1, bit-flip *s*_*Z*_; accordingly, the result becomes the same as in the case of *M*_*Z*_ = + 1 and *M*_*X*_ = + 1. Thus, Figs. 2d and e are equivalent under the feedforward to be done.

Figure 2d shows part of the circuit for the *Z*-stabilizer measurements. A similar circuit to the one in Fig. 2d but for the *X*-stabilizer measurements is shown in Fig. 2f, where |0> and |1> are respectively interchanged with |+> and |−> compared with Fig. 2d, and the roles of the control and target in CNOTs are interchanged. The state at each position is

5 $$\begin{gathered} \begin{array}{*{20}l} { \left( {\text{i}} \right){\text{:}}} \hfill & {(\alpha | + {{ > }}_{1} + \beta \left| {{-}{{ > }}_{1} )} \right| + {{ > }}_{2} \left| {0{{ > }}_{3} } \right| + {{ > }}_{4} } \hfill \\ {\left( {{\text{ii}}} \right){\text{:}}} \hfill & {(\alpha | + {{ > }}_{1} + \beta \left| {{-}{{ > }}_{1} )(} \right| + + {{ > }}_{{23}} + \left| {{-}{\text{ }}{-}{{ > }}_{{23}} )} \right| + {{ > }}_{4} } \hfill \\ {\left( {{\text{iii}}} \right){\text{:}}} \hfill & {\alpha \left( {\left| { + + + + {{ > }} + } \right| + {-}{-}{-}{{ > }}} \right) + \beta \left( {\left| {{-}{-} + + {{ > }} + } \right|{-} + {-}{-}{{ > }}} \right)} \hfill \\ {} \hfill & {\quad = (\alpha | + + {{ > }}_{{12}} + \beta \left| { - - {{ > }}_{{12}} )\;(} \right|0{{ > }}_{3} + \left| {1{{ > }}_{3} )} \right| + {{ > }}_{4} + (\alpha | + {-}{{ > }}_{{12}} + \beta \left| {{-} + {{ > }}_{{12}} )\;(} \right|0{{ > }}_{3} {-}\left| {1{{ > }}_{3} )} \right|{-}{{ > }}_{4} } \hfill \\ \end{array} \hfill \\ \begin{array}{*{20}l} { \left( {{\text{iv}}} \right){\text{:}}} \hfill & {\alpha | + + {{ > }}_{{14}} + \beta | - - {{ > }}_{{14}} } \hfill & {{\text{for}}\;\left( { + 1, + 1} \right)} \hfill \\ {} \hfill & {\alpha | + + {{ > }}_{{14}} {-}\beta | - - {{ > }}_{{14}} } \hfill & {{\text{for}}\;\left( { + 1,{\text{ }}{-}1} \right)} \hfill \\ {} \hfill & {\alpha | + {-}{{ > }}_{{14}} + \beta |{-} + {{ > }}_{{14}} } \hfill & {{\text{for}}\;\left( {{-}1,{\text{ }} + 1} \right)} \hfill \\ {} \hfill & {{-}\alpha | + {-}{{ > }}_{{14}} + \beta |{-} + {{ > }}_{{14}} } \hfill & {{\text{for}}\;\left( {{-}1,{\text{ }}{-}1} \right),} \hfill \\ \end{array} \hfill \\ \end{gathered}$$

for measured values (*M*_*X*_, *M*_*Z*_). When *M*_*Z*_ = + 1 and *M*_*X*_ = + 1, the result is equal to that for the circuit in Fig. 2g. Even if *M*_*X*_ = − 1 or *M*_*Z*_ = − 1, the result can be made to coincide with the case of *M*_*X*_ = + 1 and *M*_*Z*_ = + 1 through the feedforward. That is, Fig. 2f is equivalent to Fig. 2g under the feedforward to be done.

According to Fig. 2d–g, an equivalent CNOT is possible between data and syndrome qubits. Accordingly, stabilizer measurements are possible.

### Operation in stabilizer measurements

Adjacent *Z*- and *X*-stabilizers share two common sites; those stabilizers commute with each other in accordance with $\left[ {\hat{Z}_{a} \hat{Z}_{b} ,\hat{X}_{a} \hat{X}_{b} } \right]$ = 0, where *a* and *b* denote the common sites. The simultaneous eigenstates of the two sites are |00> ±|11> and |01> ±|10>, i.e., the Bell states. Below, we target only the two sites, because the same discussion is applicable to any pair of data-qubit sites that are nearest-neighbor in a surface code array, and we can understand the whole system with those collections.

**Figure 3:**
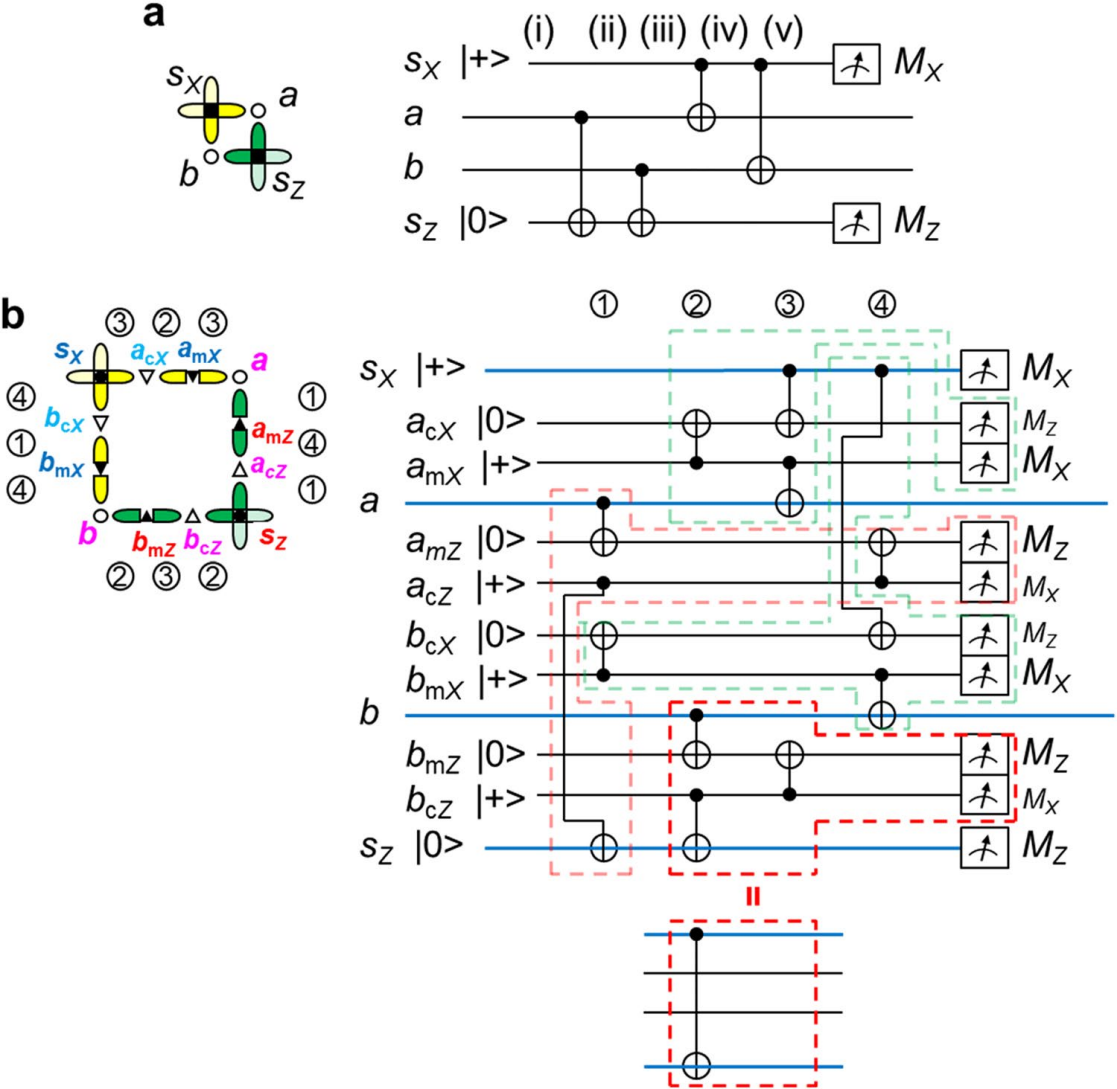
Part of a stabilizer measurement. Data qubits *a* and *b* are common between the *X*- and *Z*-stabilizers. Syndrome qubits *s*_*X*_ and *s*_*Z*_ are measured in the *X*- and *Z*-bases, respectively. (**a**) Ordinary surface code case. (**b**) Low-density qubit array case. *a*_m*Z*_, *a*_m*X*_, *b*_m*Z*_ and *b*_m*X*_ are mediator qubits; *a*_c*Z*_, *a*_c*X*_, *b*_c*Z*_ and *b*_c*X*_ are copy qubits. The circuit in (**b**) is equivalent to that in (**a**) if feedforward is done in accordance with the measurement results of mediator and copy qubits; the operation principle is based on that in Fig. 2d–g. Each area surrounded by dashed lines is equivalent to a CNOT in this sense.

Figure 3a shows part of the circuit for stabilizer measurements in the ordinary surface code focusing only on two data qubits. The left part shows the geometrical positions of data qubits *a* and *b* and related syndrome qubits *s*_*X*_ and *s*_*Z*_. The order of CNOTs is accordance with that in Fig. 1b and c. If non-commutable CNOTS are commuted, the correct outputs are not obtained^8^. As will be shown in “Methods” section, stabilizer measurements judge which Bell state the data qubits stay in (see Table 1). An error is found through a change between consecutive syndrome outputs, i.e., a change in *s*_*X*_ and *s*_*Z*_. Note that this is a change between consecutive syndrome outputs, but not |0> ↔|1> or |+> ↔|−> in Table 1.

**Table 1:** Relation between the initial and output states regarding *s*_*Z*_ and *s*_*X*_ at a stabilizer measurement.

*a*, *b*	*s*_*Z*_	*s*_*X*_
|00> +|11>	Same	Same
|00> − |11>	Same	|+> ↔|−>
|01> +|10>	|0> ↔|1>	Same
|10> − |01>	|0> ↔|1>	|+> ↔|−>

The circuit for the low-density qubit array with edge qubits is obtained by replacing CNOT with the equivalent CNOT as shown in Fig. 3b. The order of CNOTs is the same as that of ordinary stabilizer measurements, where the transformation from Fig. 2c to Fig. 2d and similar ones are used. Each part surrounded by dashed lines is equivalent to a CNOT under the feedforward to be done. Thus, the circuit in Fig. 3b is equivalent to that in Fig. 3a. Here, Fig. 3b does not have a circuit for the feedforward. This is because the feedforward is done in a software manner, as described in the next subsection.

Figure 3b means that the surface code operates even though qubits are set on edges and that the stabilizer measurements can be performed with a similar procedure as the ordinary one.

### How to handle measured values

Performing feedforward becomes another error source; it should be treated with a software process rather than with a hardware implementation.

When *M*_*Z*_ = − 1 in Fig. 2d, the bit-flipped information on *b* in Fig. 2a is transferred to *s*_*Z*_; see the third and fourth rows of Eq. (4). The same situation occurs for the information on *a*, *c* and *d* transferred to *s*_*Z*_ in Fig. 2a. Let *M*_*iZ*_ be the measured result of *i*_m*Z*_ (*i* = *a*, *b*, *c*, *d*) in Fig. 2a, and let *M*_*sZ*_ be the measured result of *s*_*Z*_. The effect of the bit flips is cancelled if *M*_*sZ*_ is multiplied by *M*_*aZ*_*M*_*bZ*_*M*_*cZ*_*M*_*dZ*_, i.e., if $M_{sZ} \prod\nolimits_{i = a}^{d} {M_{iZ} }$ is treated as the syndrome output. Real feedforward is not needed.

When *M*_*X*_ = − 1 in Fig. 2d, the phase-flipped information on *b* is transferred to *s*_*Z*_; see the second and fourth rows of Eq. (4). Because *s*_*Z*_ is measured in the *Z*-basis, the phase flip does not affect the measurement. However, when *s*_*Z*_ is measured without feedforward, *b* is phase-flipped up to a global phase because *b* and *s*_*Z*_ are entangled. This phase flip affects two *b*-related *X*-stabilizers.

In *X*-stabilizer measurements, the effects of bit and phase flips are interchanged compared with *Z*-stabilizer measurements. In Fig. 2a, let *M*_*bX*1_ and *M*_*bX*2_ be the measured results of copy qubits *b*_c*X*1_ and *b*_c*X*2_, respectively, constituting part of the *b*-related *X*-stabilizer measurements. When *M*_*bX*1_ = − 1 or *M*_*bX*2_ = − 1, *b* is bit-flipped, affecting the *b*-related *Z*-stabilizers. Here, when *M*_*bX*1_ = *M*_*bX*2_ = − 1, the effect of bit flips is cancelled. A similar situation occurs for *a*, *c* and *d*. Let *i*_c*Xj*_ (*j* = 1, 2) be two copy qubits related to *i* (*i* = *a*, *b*, *c*, *d*) for *X*-stabilizer measurements, and let *M*_*iXj*_ be the measured result of *i*_c*Xj*_. The effect of bit flips caused by measurements is correctly incorporated by defining

6 $$S_{Z} = M_{sZ} \prod\nolimits_{i = a}^{d} {\left( {M_{iZ} \prod\nolimits_{j = 1}^{2} {M_{iXj} } } \right)} ,$$

as the syndrome output for the *Z*-stabilizer $\hat{Z}_{s} = \hat{Z}_{a} \hat{Z}_{b} \hat{Z}_{c} \hat{Z}_{d}$. The syndrome output of the *X*-stabilizer $\hat{X}_{s} = \hat{X}_{a} \hat{X}_{b} \hat{X}_{c} \hat{X}_{d}$ is similarly defined, by referring to Fig. 2b, as

7 $$S_{X} = M_{sX} \prod\nolimits_{i = a}^{d} {\left( {M_{iX} \prod\nolimits_{j = 1}^{2} {M_{iZj} } } \right)} ,$$

so the effect of phase flips caused by measurements is correctly incorporated.

As described, when the measured result of a copy qubit (open triangle) is − 1, a bit or phase flip occurs. However, because those flips are tracked, the syndrome outputs are correctly obtained. In addition, those tracked flips should inevitably be reflected in a software manner in the input at the next stabilizer measurements. This procedure completes the software treatment of the feedforward.

This report concentrates only on the case where two qubits are set on edges. However, the number of qubits is not limited to two, as can be expected from the operation principle. Any even number of qubits on edges is allowed. Let us suppose that *p*-pairs of mediator and copy qubits are alternately set on edges. Then, Eqs. (6) and (7) are respectively modified into

8 $$S_{Z} = M_{sZ} \prod\nolimits_{k = 1}^{p} {\prod\nolimits_{{i = a_{k} }}^{{d_{k} }} {\left( {M_{iZ} \prod\nolimits_{j = 1}^{2} {M_{iXj} } } \right)} } ,$$

9 $$S_{X} = M_{sX} \prod\nolimits_{k = 1}^{p} {\prod\nolimits_{{i = a_{k} }}^{{d_{k} }} {\left( {M_{iX} \prod\nolimits_{j = 1}^{2} {M_{iZj} } } \right)} } ,$$

where suffix *k* is added to distinguish multiple mediator and copy qubits on each edge.

### Error rate at stabilizer measurements

Errors occur in the stabilizer measurements during initialization and measurements, at CNOTs, and during idle time. However, we cannot generally distinguish them. In the following, we treat errors during the idle time as part of other errors and thereby omit the classification of idle time. In addition, we will set aside consideration of errors at Hadamard gates needed in the *X*-stabilizer measurements for simplicity; a Hadamard gate is needed when the initialization state is |+> = $\hat{H}\left| 0 \right\rangle$ and when a measurement is done in the *X*-basis.

The following is a rough estimate. Let *p*_C*X*_ be the error rate at CNOTs, *p*_*i*_ be that at the initializations of qubits, and *p*_*d*_ be that at the measurements of the qubits. When stabilizer measurements are performed in accordance with the circuit in Fig. 1b and c, the error rate during one round of stabilizer measurements is *p*_*i*_ + 4*p*_C*X*_ + *p*_*d*_ ≡ *p*_or_ if *p*_*i*_ , *p*_C*X*_, *p*_*d*_ << 1.

In the low-density qubit array case, the syndrome output is obtained by multiplying the measured values of the mediator, copy, and syndrome qubits in accordance with Eqs. (6) and (7). The measured value of a mediator qubit contributes to one syndrome output according to Eqs. (6) and (7); the measured value of a copy qubit contributes to two syndrome outputs. Let us suppose that an error has occurred at a measurement for *b*_c*Z*_ in Fig. 2a. The result affects two *b*-related *X*-stabilizer outputs. In this case, we cannot judge whether the measurement error is at *b*_c*Z*_ or an error in *b* itself. However, this error judgement is made after *d* rounds of stabilizer measurements, as will be described in “Methods” section. When an error signal appears as pairs on the time axis, the error is judged to be a measurement error at *b*_c*Z*_ (see “Methods” section). When no pairs appears on the time axis, it is judged to be an error in *b*. Because the syndrome output is the product of the measured values, each of which has no meaning in itself, we generally cannot judge where an error occurs in stabilizer measurements. However, the measurement error at *b*_c*Z*_ can be identified, as described above.

In the low-density qubit array case, three CNOTs are used to achieve an equivalent CNOT, as shown in Fig. 2d and f. A stabilizer measurement uses twelve CNOTs because four data qubits are involved. Let us focus our attention on the uppermost CNOT in Fig. 2d, for example. Although the CNOT does not directly operate on the syndrome qubit, because the syndrome and data qubits are entangled through two other CNOTs, the CNOT affects the syndrome qubit. That is, all twelve CNOTs can cause errors in the stabilizer measurements. Their contributions to the error rate total 12*p*_C*X*_. Initializations at stabilizer measurements are done for four mediator, four copy, and one syndrome qubit, e.g., inside the dashed circle of Z_*s*_ in Fig. 2a. If the initialization of these nine qubits is incomplete, it affects the result. Thus, its contribution to the error rate is 9*p*_*i*_. Because the measured values used for a syndrome output are those of four mediator, eight copy, and one syndrome qubits, their contribution is 13*p*_*d*_. Summing these errors gives 9*p*_*i*_ + 12*p*_C*X*_ + 13*p*_*d*_ ≡ *p*_ff_. For simplicity, let *p*_ave_ ≃ *p*_*i*_, *p*_C*X*_, *p*_*d*_; then *p*_ff_ ≃ 34*p*_ave_. In the case of ordinary surface code, the same simplification gives *p*_or_ ≃ 6*p*_ave_. Therefore, when two qubits are set on edges, the error rate needs to be lowered to 6/34 ≃ 1/6th that of ordinary surface code on average. This is one of drawbacks of the low-density qubit array. We expect that future progress in the error rate reduces this problem.

### Error rate of data qubits

Data qubits might have errors during logical-qubit operations, during idle time, and also during stabilizer measurements. The difference between ordinary surface code and the low-density qubit array case exists in the stabilizer measurements. Let us focus our attention on this difference.

A data qubit interacts with four syndrome qubits through a CNOT during one round of stabilizer measurements in the ordinary surface code. The error rate per CNOT is *p*_C*X*_ ≃ *p*_ave_.

The equivalent CNOT in the low-density qubit array case is achieved using initialized mediator and copy qubits and three CNOTs, as shown in Fig. 2d and f. All these elements can cause an error in the equivalent CNOT. Thus, the error rate per equivalent CNOT is estimated to be 3*p*_C*X*_ + 2*p*_*i*_ ≃ 5*p*_ave_. This means that the error rate is five times that of ordinary surface code. According to the discussion in the preceding subsection, the average error rate must be ~ 1/6th that of ordinary surface code. The requirement in this subsection is 1/5, which is the same level as ~ 1/6.

### Logical qubits

**Figure 4:**
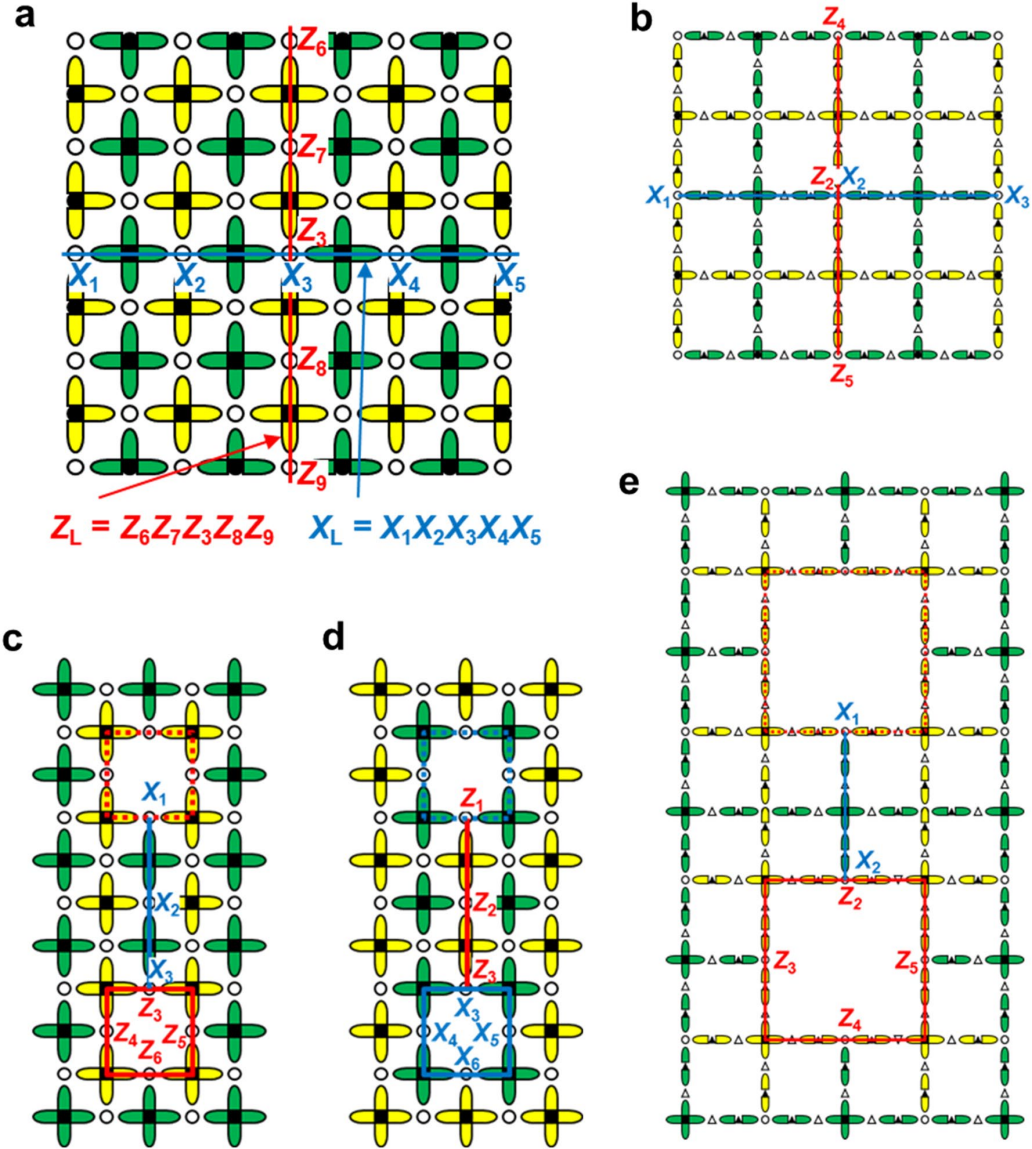
Logical *X* and *Z* operators. (**a**,**b**) They are defined by connecting boundaries. (**c**–**e**) Defect-based logical qubits. (**a**, **c** and **d**) are the case of the ordinary surface code. (**b**) and (**e**) are the case of the low-density qubit array case. (**c**) and (**e**) show *Z*-cut logical qubits formed by turning off two syndrome-*Z* qubits. (**d**) shows an *X*-cut logical qubit formed by turning off two syndrome-*X* qubits.

As will be described in “Methods” section in detail, the logical *X*-operator is defined as $\hat{X}_{{\text{L}}}$ = $\hat{X}_{1} \hat{X}_{2} \hat{X}_{3} \hat{X}_{4} \hat{X}_{5}$ in Fig. 4a by connecting two *X*-boundaries (comprised of yellow edges) in the ordinary surface code. Similarly, the logical *Z*-operator is defined as $\hat{Z}_{{\text{L}}} = \hat{Z}_{6} \hat{Z}_{7} \hat{Z}_{3} \hat{Z}_{8} \hat{Z}_{9}$ by connecting two *Z*-boundaries (comprised of green edges),where *d* = 5 (see “Methods” section as for *d*). The low-density qubit array case is also similar: $\hat{X}_{{\text{L}}}$≡$\hat{X}_{1} \hat{X}_{2} \hat{X}_{3}$ and $\hat{Z}_{{\text{L}}} \equiv \hat{Z}_{4} \hat{Z}_{2} \hat{Z}_{5}$ in Fig. 4b, where *d* = 3.

More logical qubits can be defined by adding boundaries. Boundaries can be artificially built by introducing defects, as shown in Fig. 4c, where a *Z*-stabilizer measurement is turned off (*Z*-cut qubit), thereby an *X*-boundary is built inside the array. The logical operators are defined using a pair of defects as $\hat{X}_{{\text{L}}}$≡$\hat{X}_{1} \hat{X}_{2} \hat{X}_{3}$ and $\hat{Z}_{{\text{L}}} \equiv \hat{Z}_{3} \hat{Z}_{4} \hat{Z}_{5} \hat{Z}_{6}$ with *d* = 3. Figure 4d shows the case where a pair of *X*-stabilizer measurements is turned off (*X*-cut qubit). The logical operators are defined as $\hat{Z}_{{\text{L}}} \equiv \hat{Z}_{1} \hat{Z}_{2} \hat{Z}_{3}$ and $\hat{X}_{{\text{L}}}$≡$\hat{X}_{3} \hat{X}_{4} \hat{X}_{5} \hat{X}_{6}$. The low-density qubit array case is also similar: $\hat{X}_{{\text{L}}}$≡$\hat{X}_{1} \hat{X}_{2}$ and $\hat{Z}_{{\text{L}}} \equiv \hat{Z}_{2} \hat{Z}_{3} \hat{Z}_{4} \hat{Z}_{5}$ in Fig. 4e, where *d* = 2.

Boundaries can also be increased by using a method called lattice surgery^19^. This is also applicable to the low-density qubit array case.

## Discussion

**Figure 5:**
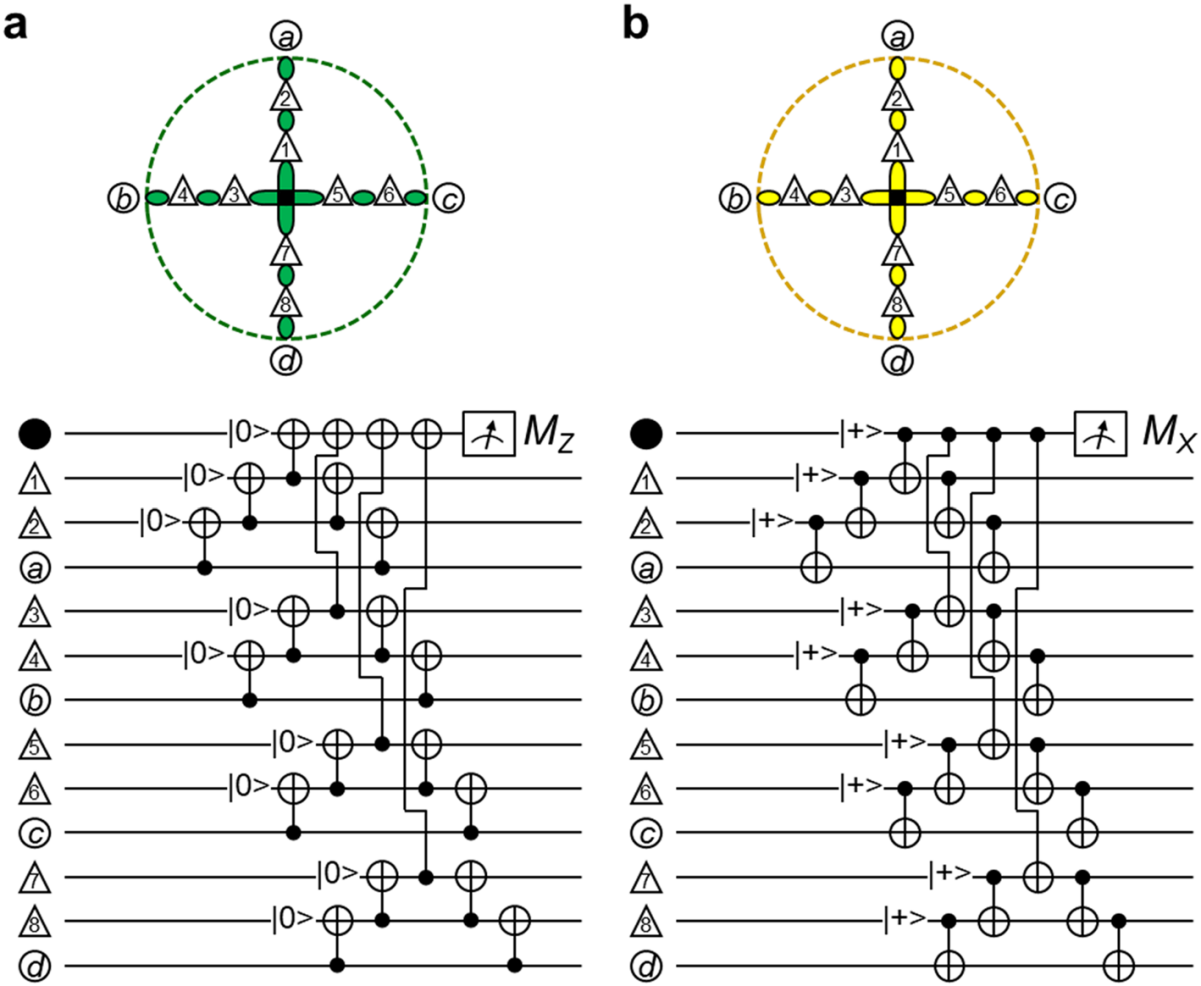
Circuits for a simple method of stabilizer measurements. CNOTs are repeated to transfer the information on data qubits to a syndrome qubit. (**a**) Circuit for detecting a bit-flip error. (**b**) Circuit for detecting a phase-flip error.

The low-density qubit array case in this report transfers the information on data qubits to syndrome qubits by measuring qubits on edges and performing feedforward. However, this is not a unique method. The information transfer can also be done by repeating CNOTs. Figure 5a and b show the circuits for *Z*- and *X*-stabilizer measurements. The information on data qubits *a*, *b*, *c* and *d* are transferred to the syndrome qubit (solid circle) by repeating CNOTs; after the transfer, CNOTs are again repeated to lift the entanglement. One round of stabilizer measurements is within six steps if the timing of the initialization is shifted qubit by qubit. Here, because *X*-stabilizer measurements initialize syndrome and edge qubits with |+> = $\hat{H}\left| 0 \right\rangle$ and measure them in the *X*-basis, Hadamard gates are needed; thus, the step count is eight. The number of steps is the same as that of the ordinary surface code and that of the feedforward type.

There are twenty CNOTs in Fig. 5. As for the feedforward type, there are 12 CNOTs in one round of stabilizer measurements. The number of measurements is one in Fig. 5 and nine for the feedforward type. Those numbers are summarized in Table 2. Which method is better depends on the level of difficulty regarding the CNOT and the measurement. If the measurement is easier than the CNOT, the feedforward type is advantageous; if the CNOT is easier than the measurement, the simple method is advantageous. However, we cannot simply determine which is easier. Let us consider this situation from the viewpoint of fidelity (error rate). The difficulties regarding the CNOT and the measurement are currently at the same level, e.g., in superconductor quantum computers^20–22^, in ion-trap ones^20^, in nuclear-spin semiconductor ones^23^, and in electron-spin semiconductor ones^24–29^. Here, when the CNOT is not the native gate, the fidelity (error rate) can be estimated from the native two-qubit gate and single-qubit gate.

**Table 2:** Number of operations in one round of stabilizer measurements, and error rate.

	Initialization	CNOT	Measurement	step	@Stabilizer measurement	@CNOT equivalent
Ordinary	1	4	1	6 (8)	*p*_*i*_ + 4*p*_C*X*_ + *p*_*d*_ ≃ 6*p*_ave_	*p*_C*X*_ ≃ *p*_ave_
Feedforward	9	12	9	6 (8)	9*p*_*i*_ + 12*p*_C*X*_ + 13*p*_*d*_ ≃ 34*p*_ave_	3*p*_C*X*_ + 2*p*_*i*_ ≃ 5*p*_ave_
Simple	9	20	1	6 (8)	9*p*_*i*_ + 20*p*_C*X*_ + *p*_*d*_ ≃ 30*p*_ave_	5*p*_C*X*_ ≃ 5*p*_ave_

The bracket in the “step” column indicates the *X*-stabilizer case.

The right-most column indicates the error rate regarding data qubits at one equivalent CNOT.

Next, let us estimate the error rate of the simple method by assuming *p*_ave_ ≃ *p*_*i*_, *p*_C*X*_, *p*_*d*_, similarly to the estimation for the feed-forward type. Regarding the stabilizer measurements, the number of CNOTs is twenty, contributing an error rate of 20*p*_C*X*_. Here, the reason why the CNOTs for lifting the entanglement are taken into account is that if the lifting is insufficient because of a CNOT error, the effect of the edge qubits on the syndrome qubit remains; in this case, the state of data and syndrome qubits becomes a mixed state after the partial trace with respect to the edge qubits; thus, the CNOTs for the lifting affects the syndrome qubit. The initialization of eight edge qubits and one syndrome qubit amounts to 9*p*_*i*_. Only the syndrome qubit is measured, contributing *p*_*d*_. Their sum is 9*p*_*i*_ + 20*p*_C*X*_ + *p*_*d*_ ≡ *p*_si_. If *p*_ave_ ≃ *p*_*i*_, *p*_C*X*_, *p*_*d*_, then *p*_si_ ≃ 30*p*_ave_.

The feedforward type gave *p*_ff_ ≃ 34*p*_ave_. Therefore, if the error rates of the measurement and CNOT are at the same level, there is no substantial difference between the feedforward and simple methods. The advantages of the feedforward type are that the timings of the initializations, gates, and measurements are synchronized, as shown in Fig. 3b and that the stabilizer measurement procedure is similar to that of ordinary surface code, except for the feedforward in a software manner being added. In contrast, the timings are not synchronized in the simple method. The initializations, gates, and measurements are different in terms of their operation and duration. Synchronization in the feedforward type is thus a remarkable characteristic from the viewpoint of parallel executions.

The second estimate is the error rate of the data qubits. The information transfer from a data qubit to a syndrome qubit uses one CNOT in ordinary surface code. The simple method in Fig. 5 uses five CNOTs for that purpose. The error rate is estimated as 5*p*_C*X*_. In contrast to the feedforward type, initialization errors on edge qubits do not affect data qubits in terms of the first-order error because the edge qubits are disentangled from the data qubits at the input and output of the edge qubits. Because the error rate for ordinary surface code is *p*_C*X*_, the error rate needs to be 1/5th that of ordinary surface code; this is of the same level as in the stabilizer measurements.

In this section, we compared the feedforward and simple methods and found that we cannot simply say which is better. It depends on the design policy and the difficulty level of the measurement and CNOT. However, the synchronized timing characteristic of the feedforward type is advantageous. In addition, it is advantageous that the feedforward type and the ordinary surface code share the same stabilizer measurement procedure; the only difference is the software part. This similarity gives us freedom in designing qubit arrays.

## Methods

### Logical qubits

Even though qubits are set on the edges, the surface code can operate similarly to the ordinary case if the measured values of the syndrome, mediator, and copy qubits are appropriately treated as described so far. This subsection summarizes important characteristics of logical qubits using the ordinary surface code.

Figure 4a consists of 41 data qubits and 40 syndrome qubits. The number of data qubits exceeds that of the syndrome qubits by one. The difference corresponds to the freedom of a logical qubit. As described in the preceding section, the logical *X* and *Z* operators are defined by connecting two *X*- and *Z*-boundaries, respectively. If the connection rule is satisfied, the logical qubit may be geometrically deformed^8^. The distance *d* is defined to be the minimum under the rule, and *d* = 5 in Fig. 4a. In defect qubits, the distance can be increased by moving the defects apart and by enlarging the defect size. The positions of the logical qubits can be moved through geometrical transformation.

The common site between $\hat{X}_{{\text{L}}}$ and $\hat{Z}_{{\text{L}}}$ in Fig. 4a is only site 3. That means $[ {\hat{Z}_{{\text{L}}} ,\hat{X}_{{\text{L}}} } ]$ = $[ {\hat{Z}_{{3}} ,\hat{X}_{{3}} } ]$; thus, $\hat{X}_{{\text{L}}}$ and $\hat{Z}_{{\text{L}}}$ satisfy the ordinary commutation relation. The blue line in Fig. 4a visualizes the $\hat{X}_{{\text{L}}}$. A green cross visualizes a *Z*-stabilizer. Each *Z*-stabilizer under the blue line includes two of $\hat{Z}_{1}$,$\hat{Z}_{2}$,$\hat{Z}_{3}$,$\hat{Z}_{4}$ and $\hat{Z}_{5}$. Therefore, those stabilizers and $\hat{X}_{{\text{L}}}$ commute. The other *Z*-stabilizers do not include $\hat{Z}_{1}$,$\hat{Z}_{2}$,$\hat{Z}_{3}$,$\hat{Z}_{4}$ and $\hat{Z}_{5}$, and thus commute with $\hat{X}_{{\text{L}}}$. The *X*-stabilizers and $\hat{X}_{{\text{L}}}$ trivially commute. Thus, $\hat{X}_{{\text{L}}}$ commutes with all stabilizers. Similarly, $\hat{Z}_{{\text{L}}}$ commute with all stabilizers. These commutabilities assure that the stabilizer measurements do not affect the logical qubit.

### Universal gates

The logical CNOT can be performed by braiding a *Z*-cut qubit and an *X*-cut qubit using geometrical transformation^8^. The logical *S*_L_ (= *Z*_L_^1/2^) gate is possible using an ancilla qubit prepared in a specific state; the logical *T*_L_ (= *S*_L_^1/2^) gate can be realized further with the addition of a feedforward technique^8^. The logical Hadamard gate *H*_L_ can be achieved by a combination of geometrical transformation, data-qubit-Hadamard gates, and SWAPs between physical qubits^8^. These gates constitute the minimum elements for universal quantum computing.

Because the Hadamard gate interchanges *X* and *Z* operators, logical *X*_L_ and *Z*_L_ operators are also interchanged. In Fig. 4a, the blue (red) line visualizing $\hat{X}_{{\text{L}}} ( {\hat{Z}_{{\text{L}}} } )$ runs along the green (yellow) crosses visualizing *Z*-stabilizers (*X*-stabilizers). However, when $\hat{X}_{{\text{L}}}$ and $\hat{Z}_{{\text{L}}}$ are interchanged, the blue and red lines are interchanged; then, the blue (red) line runs along the yellow (green) crosses visualizing *X*-stabilizers (*Z*-stabilizers). This situation is that the blue (red) line is 90-degree-rotated and lies on the wrong row and column. The rotation is cancelled by a geometrical rotation. The incorrect lying is compensated by displacing $\hat{X}_{{\text{L}}} ( {\hat{Z}_{{\text{L}}} } )$ by one row and one column by repeating SWAP gates in units of row and column; the row SWAPs are done in series from the top to bottom rows; the column SWAPs are done in series from the left to the right columns. The displacement achieved by the repeated SWAPs is easily understood in the case of the two-dimensional grid array. However, when qubits are set also on edges, it is not so easily understood because qubits on row (column) edges cannot move in the column (row) direction. Nevertheless, displacing $\hat{X}_{{\text{L}}} ( {\hat{Z}_{{\text{L}}} } )$ is possible.

**Figure 6:**
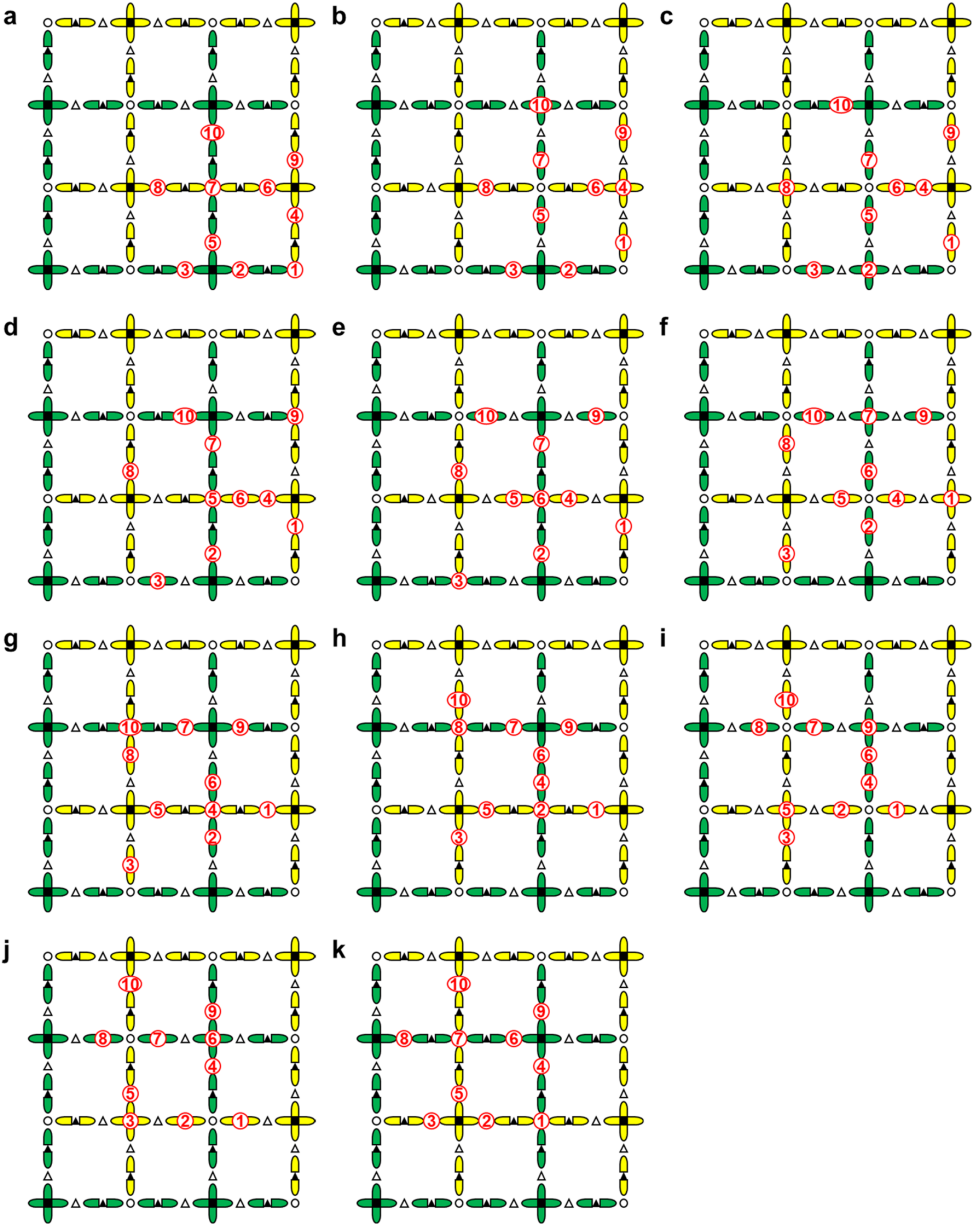
Procedure for the parallel displacement included in $\hat{H}_{{\text{L}}}$. ①–⑩ depict data qubits in a primitive cell. (**a**) Initial state. (**b**,**d**,**f**,**h**,**j**) Physical qubits on vertical edges and on nodes of the grid are vertically displaced by one step. The displacement is achieved using swap gates in units of row. (**c**,**e**,**g**,**i**,**k**) Physical qubits on horizontal edges and on nodes of the grid are horizontally displaced by one step. The displacement is achieved using swap gates in units of column. (**k**) As a result of the displacements, the data qubits are displaced from the initial state by half the primitive cell. There are intervals during these swap processes when error detection measurements cannot be performed. In (**f**), the positions of the syndrome qubits and the data qubits are temporarily interchanged.

Figure 6 can help us to understand the situation visually. Figure 6a is the initial state. First, each qubit is vertically displaced by one step by repeating swap gates in units of row, where the qubits on the row edges stay at their sites. Figure 6b is the result. While qubits 1, 4, 5, 7, 9, and 10 are displaced by one step, qubits 2, 3, 6, and 8 stay at their sites. Next, as shown in Fig. 6c, the qubits are horizontally displaced by one step, where qubits 2 and 8 have moved to grid nodes. Then, they can move vertically as shown in Fig. 6d. We mentioned only qubits 2 and 8 here, but all qubits have opportunities to move vertically and horizontally according to the procedure shown in Fig. 6, where row and column displacements are alternately repeated as shown in Fig. 6a–k. Thus, one-row and one-column displacements can be made on the two-dimensional grid including edge qubits; the configuration in Fig. 6k is just one-row and one-column shift from that in Fig. 6a. Thus, a logical Hadamard gate is possible even though qubits are on the edges. Here, the number of SWAPs needed is three times that for ordinary surface code.

### How to judge an error

Syndrome outputs are given by Eqs. (6) and (7) when qubits are set on the edges. How to judge an error is the same as that in the ordinary surface code. In the following, we describe the basic principle by referring to Fig. 1a expressing the ordinary surface code.

As an example, let us suppose that the outputs of *s*1 and *s*3 in Fig. 1a change between consecutive measurements, and that other outputs do not change. This result suggests a bit flip occurs at qubit 3. As can be understood from this example, an error in a data qubit causes a pair of output changes, although boundaries are an exception^8,30^. A phase-flip error on a *Z*-boundary (comprised of green edges) causes an output change only for a single *X*-stabilizer; a bit-flip error on an *X*-boundary (comprised of yellow edges) causes an output change only for a single *Z*-stabilizer. Errors also occur in stabilizer measurements. In this case, an output change occurs just after the error and in the next measurement as well because there is no error in the next output. That is, the error takes the form of a pair of changes on the time axis. Thus, judging errors is equivalent to identifying pairs, except boundaries, in a three-dimensional space consisting of space and time axes. Pairs can be identified with a maximum likelihood approach; a minimum-weight perfect-matching algorithm is used^31–33^. The minimum length for the logical qubit is *d* = 5 in Fig. 4a. Therefore, the search space for the two-dimensional surface is *d* × *d*. When the error rates of the qubits and stabilizer measurements are comparable, the search space for the time axis is also around *d*. That is, an error judgement is made after *d* stabilizer measurements^8,30^.

### Stabilizer states

Adjacent *Z*- and *X*-stabilizers share two common sites; the simultaneous eigenstates of the two sites are |00> ±|11> and |01> ±|10>. We concretely show that the simultaneous eigen states are held through stabilizer measurements and that the states can be judged through the output of stabilizer measurements. The whole system is understood as those collections. The principle is also the same for the low-density qubit array case; therefore, we describe it by referring to the ordinary surface code.

The left part of Fig. 3a shows the geometrical positions of data qubits *a* and *b* and related syndrome qubits *s*_*X*_ and *s*_*Z*_. Let the initial state of *s*_*X*_ be |+>, the initial state of *s*_*Z*_ be |0>, and the input state of *a* and *b* be |00> ±|11>. Then, the input for the circuit in Fig. 3a is

10 $$\left( {\text{i}} \right){:}\quad \left| { + {>} (} \right|00 {{>} \pm} \left| {{11} {>} )} \right|0 {>} \; = \left| { + {>} } \right|0 {>} \left| {0 {>} } \right|0 {{>} \pm} \, \left| { + {>} } \right|{1} {>} \left| {{1} {>} } \right|0 {>} .$$

Here, the kets denote the states of *s*_*X*_, *a*, *b*, and *s*_*Z*_ in this order. The first CNOT in the circuit of Fig. 3a operates with *a* being the control and *s*_*Z*_ being the target; the input state evolves to

11 $$\left( {{\text{ii}}} \right){:}\quad \left| { + {>} } \right|0 {>} \left| {0 {>} } \right|0 {{>} \pm} \left| { + {>} } \right|{1} {>} \left| {{1} {>} } \right|{1} {>} .$$

At the second CNOT, *b* is the control and *s*_*Z*_ the target; the state evolves to

12 $$\left( {{\text{iii}}} \right){:}\quad \left| { + {>} } \right|0 {>} \left| {0 {>} } \right|0 {> \pm} \left| { + {>} } \right|{1} {>} \left| {{1} {>} } \right|0 {>} .$$

At the third CNOT, *s*_*X*_ is the control and *a* the target. Let us rewrite |+> as |0> +|1>. The state evolves to

13 $$\left( {{\text{iv}}} \right){:}\quad \left( {\left| {00 {>} + } \right|{11} {>} } \right)\left| {0 {>} } \right|0 {> \pm} \left( {\left| {0{1} {>} + } \right|{1}0 {>} } \right)\left| {{1} {>} } \right|0 {>} .$$

At the fourth CNOT, *s*_*X*_ is the control and *b* the target; the state evolves to

14 $$\begin{aligned} \left( {\text{v}} \right){:}\quad & \left( {\left| {000 {>} + } \right|{111} {>} } \right)\left| {0 {{>} \pm} \;(} \right|0{11} {>} + \left| {{1}00 {>} )} \right|0 {>} \\ & \quad = \left( {\left| {0 {{>} \pm} } \right|{1} {>} } \right)\left| {00 {>} } \right|0 {>} + \left( {\left| {{1} {{>} \pm} } \right|0 {>} } \right)\left| {{11} {>} } \right|0 {>} \\ & \quad = \left| { {\pm {>}} (} \right|00 {{>} \pm} \left| {{11} {>} )} \right|0 {>} . \\ \end{aligned}$$

The output state of *a* and *b* is |00> ±|11>, which is the same as the input state. Because the states of *s*_*X*_ and *s*_*Z*_ are disentangled from those of *a* and *b*, stabilizer measurements do not affect the state of *a* and *b*. The output state of *s*_*Z*_ is |0>, meaning that the state of *s*_*Z*_ is maintained. The output state of *s*_*X*_ is |+> when the state of *a* and *b* is |00> +|11> and |−> when the state of *a* and *b* is |00> − |11>. The former case means the state of *s*_*X*_ is maintained; the latter case means the state of *s*_*X*_ is phase-flipped.

We can replace |+> with |−> for the initial state of *s*_*X*_, and |0> with |1> for the initial state of *s*_*Z*_. A similar analysis as above shows that the output state of *s*_*Z*_ is |1>, i.e., that the state of *s*_*Z*_ is maintained. The output state of *s*_*X*_ is |−> when the state of *a* and *b* is |00> +|11> and |+> when the state of *a* and *b* is |00> − |11>, i.e., the state of *s*_*X*_ is maintained in the former case and phase-flipped in the latter case. The summary is that the state of *s*_*X*_ is phase-flipped when the state of *a* and *b* is |00> − |11>; the other cases maintain the states of *s*_*X*_ and *s*_*Z*_; Table 1 summarizes all cases.

The case in which the input state of *a* and *b* is |01> ±|10> is also treated similarly, i.e.,

15 $$\begin{aligned} \left( {\text{i}} \right){:}\quad & \left| { {+ >} (} \right|0{1} {> \pm} \left| {{1}0 {>} )} \right|0 {>} \; = \left| { + {>} } \right|0 {>} \left| {{1} {>} } \right|0 {{>} \pm} \left| { + {>} } \right|{1} {>} \left| {0 {>} } \right|0 {>} \\ \left( {{\text{ii}}} \right){:}\quad & \left| { + {>} } \right|0 {>} \left| {{1} {>} } \right|0 {{>} \pm} \left| { + {>} } \right|{1} {>} \left| {0 {>} } \right|{1} {>} \\ \left( {{\text{iii}}} \right){:}\quad & \left| { + {>} } \right|0 {>} \left| {{1} {>} } \right|{1} {{>} \pm} \left| { + {>} } \right|{1} {>} \left| {0 {>} } \right|{1} {>} \\ \left( {{\text{iv}}} \right){:}\quad & \left( {\left| {00 {>} + } \right|{11} {>} } \right)\left| {{1} {>} } \right|{1} {{>} \pm} \left( {\left| {0{1} {>} + } \right|{1}0 {>} } \right)\left| {0 {>} } \right|{1} {>} \\ \left( {\text{v}} \right){:}\quad & \left( {\left| {00{1} {>} + } \right|{11}0 {>} } \right)\left| {{1} {{>} \pm} \;(} \right|0{1}0 {>} + \left| {{1}0{1} {>} )} \right|{1} {>} \\ & \quad = \left( {\left| {0 {{>} \pm} } \right|{1} {>} } \right)\left| {0{1} {>} } \right|{1} {>} + \left( {\left| {{1} {{>} \pm} } \right|0 {>} } \right)\left| {{1}0 {>} } \right|{1} {>} \\ & \quad = \left| { {\pm {>}} (} \right|0{1} {{>} \pm} \left| {{1}0 {>} )} \right|{1} {>} . \\ \end{aligned}$$

Here, the state of *s*_*Z*_ is bit-flipped. The state of *s*_*X*_ is respectively maintained and phase-flipped when the state of *a* and *b* is |01> +|10> and |01> − |10> (see Table 1).

As is apparent from Table 1, we can distinguish the state of the data qubits from the outputs of *s*_*X*_ and *s*_*Z*_. Because any two-qubit state can be expanded with the Bell states, Eqs. (10)–(15) complete the analysis of the dynamics involved in the stabilizer measurements.

## Data Availability

The datasets used during the current study are available from the corresponding author on reasonable request.
